# The Contribution of Genetic Risk and Lifestyle Factors in the Development of Adult-Onset Inflammatory Bowel Disease: A Prospective Cohort Study

**DOI:** 10.14309/ajg.0000000000002180

**Published:** 2023-01-09

**Authors:** Yuhao Sun, Shuai Yuan, Xuejie Chen, Jing Sun, Rahul Kalla, Lili Yu, Lijuan Wang, Xuan Zhou, Xiangxing Kong, Therese Hesketh, Gwo-tzer Ho, Kefeng Ding, Malcolm Dunlop, Susanna C. Larsson, Jack Satsangi, Jie Chen, Xiaoyan Wang, Xue Li, Evropi Theodoratou, Edward L. Giovannucci

**Affiliations:** 1 Department of Big Data in Health Science, School of Public Health and The Second Affiliated Hospital, Zhejiang University School of Medicine, Hangzhou, China;; 2 Centre for Global Health, School of Public Health, Zhejiang University School of Medicine, Hangzhou, China;; 3 Unit of Cardiovascular and Nutritional Epidemiology, Institute of Environmental Medicine, Karolinska Institutet, Stockholm, Sweden;; 4 Department of Gastroenterology, The Third Xiangya Hospital, Central South University, Changsha, China;; 5 Department of Gastroenterology, Royal Infirmary of Edinburgh, University of Edinburgh, Edinburgh, UK;; 6 Centre for Global Health, Usher Institute, University of Edinburgh, Edinburgh, UK;; 7 Department of Colorectal Surgery and Oncology, Key Laboratory of Cancer Prevention and Intervention, Ministry of Education, The Second Affiliated Hospital, Zhejiang University, Hangzhou, China;; 8 Institute for Global Health, University College London, London, UK;; 9 Edinburgh IBD Science Unit, Centre for Inflammation Research, University of Edinburgh, Edinburgh, UK;; 10 Cancer Research UK Edinburgh Centre, Medical Research Council Institute of Genetics and Cancer, University of Edinburgh, Edinburgh, UK;; 11 Unit of Medical Epidemiology, Department of Surgical Sciences, Uppsala University, Uppsala, Sweden;; 12 Translational Gastroenterology Unit, Nuffield Department of Medicine, Experimental Medicine Division, University of Oxford, John Radcliffe Hospital, Oxford, UK;; 13 Department of Epidemiology, Harvard T.H. Chan School of Public Health, Boston, Massachusetts, USA;; 14 Department of Nutrition, Harvard T.H. Chan School of Public Health, Boston, Massachusetts, USA.

**Keywords:** inflammatory bowel disease, Crohn's disease, ulcerative colitis, lifestyle, polygenic risk score

## Abstract

The joint associations across genetic risk, modifiable lifestyle factors, and inflammatory bowel disease (IBD) remains unclear.

**METHODS:**

Genetic susceptibility to Crohn's disease (CD) and ulcerative colitis (UC) was estimated by polygenic risk scores and further categorized into high, intermediate, and low genetic risk categories. Weighted healthy lifestyle scores were constructed based on 5 common lifestyle factors and categorized into favorable (4 or 5 healthy lifestyle factors), intermediate (3 healthy lifestyle factors), and unfavorable (0–2 healthy lifestyle factors) groups. Cox proportional hazards regression model was used to estimate the hazard ratios (HR) and 95% confidence interval (CI) for their associations.

**RESULTS:**

During the 12-year follow-up, 707 cases with CD and 1576 cases with UC were diagnosed in the UK Biobank cohort. Genetic risk and unhealthy lifestyle categories were monotonically associated with CD and UC risk with no multiplicative interaction between them. The HR of CD and UC were 2.24 (95% CI 1.75–2.86) and 2.15 (95% CI 1.82–2.53) for those with a high genetic risk, respectively. The HR of CD and UC for individuals with an unfavorable lifestyle were 1.94 (95% CI 1.61–2.33) and 1.98 (95% CI 1.73–2.27), respectively. The HR of individuals with a high genetic risk but a favorable lifestyle (2.33, 95% CI 1.58–3.44 for CD, and 2.05, 95% CI 1.58–2.66 for UC) were reduced nearly by half, compared with those with a high genetic risk but an unfavorable lifestyle (4.40, 95% CI 2.91–6.66 for CD and 4.44, 95% CI 3.34–5.91 for UC).

**DISCUSSION:**

Genetic and lifestyle factors were independently associated with susceptibility to incident CD and UC. Adherence to a favorable lifestyle was associated with a nearly 50% lower risk of CD and UC among participants at a high genetic risk.

## Graphical abstract

**Figure:**
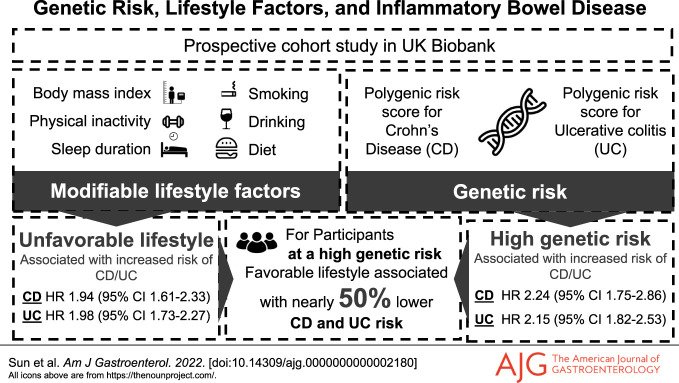

## INTRODUCTION

Inflammatory bowel disease (IBD), which includes 2 main subtypes (Crohn's disease [CD], and ulcerative colitis [UC]), is a global health problem with substantial disease burden, especially in the industrialized countries (1,2). Although onset in childhood and early adulthood is well recognized, epidemiological studies now highlight the increasing incidence and prevalence of IBD onset in middle age or later life. Compared with IBD in children or adolescents, the etiology of adult-onset IBD is believed to be more multifactorial, with genetic and environmental factors playing important roles in its development (3,4).

Genome-wide association studies have identified more than a 100 of risk loci, such as *TYK2*, *IL2RA*, and *IL23R*, to be associated with IBD (3,5–9). Although a single genetic variant accounts for only a small fraction of the genetic variability of IBD, polygenic risk scores combining multiple risk loci can be used as an indicator to identify individuals at higher genetic susceptibility to IBD (10). Compared with rare genetic mutations with larger effect (e.g., *NOD2*), polygenic risk scores can identify a larger fraction of population at comparable or greater disease risk, which poses opportunities for clinical utility. However, it is largely unknown whether a polygenetic risk score (PRS) of IBD can identify individuals at a high genetic risk for potential personalized prevention through adoption of healthy lifestyles in later life.

Observational studies have identified several potentially modifiable risk factors in relation to IBD, including cigarette smoking, unhealthy diet, physical inactivity, obesity, and abnormal sleep duration (4,11). The associations of these lifestyle factors with the risk of CD and UC seem complex. For instance, active smoking has been reported to be protective against UC but risky for CD (11). The association between alcohol drinking and IBD is inconclusive and remains elusive (12). Sleep is one of the common lifestyle factors in maintaining physical and psychological health; however, whether sleep behavior is associated with IBD risk has been scarcely investigated (13). Comprehensive appraisal of the associations between these modifiable lifestyle factors and IBD risk will deepen the understanding of the etiology of IBD and provide clues for IBD prevention.

So far, there is a lack of comprehensive investigation on the combined effect of genetic and lifestyle factors on the development of IBD and its subtypes. We conducted a prospective cohort study based on the UK Biobank to examine the associations across genetic risk, modifiable lifestyle factors, and risk of IBD to test whether there is any multiplicative interaction between genetic risk and lifestyle factors and to figure out to what extent the genetic risk of IBD may be mitigated by adherence to healthy lifestyle choices.

## METHODS

### Study population

**Figure 1.**
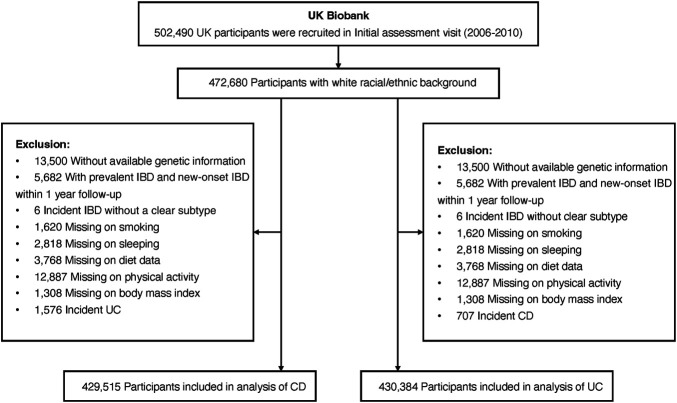
Flowchart of study population selection in the UK Biobank study. CD, Crohn's disease; IBD, inflammatory bowel disease; UC, ulcerative colitis.

This cohort study is based on data collected from the UK Biobank including approximately 500,000 participants recruited across the United Kingdom between 2006 and 2010 (14). Individuals of non-European ancestry (due to limited numbers and to minimize population structure bias), without genetic information, or with baseline IBD diagnosis, new-onset IBD within 1-year follow-up (to minimize reverse causality), or unclear IBD diagnosis were excluded, leaving 453,492 individuals (Figure 1).

### Genetic risk profiling

We applied 2 strategies to estimate the genetic susceptibility to CD and UC for the UK Biobank population. We first constructed a PRS using the common genetic variants that were identified to be strongly associated with CD and UC (*P* < 5 × 10^−8^) from a genome-wide association meta-analysis of up to 86,640 individuals of European ancestry, including 5,956 cases with CD and 6,968 cases with UC (3). After removing genetic variants in linkage disequilibrium, 51 and 30 independent single-nucleotide polymorphisms (SNP) (*r*^2^ < 0.001) were used to calculate the PRS of CD and UC, respectively. Polygenic risk scores were constructed for each participant by summing up the number of risk-increasing alleles for each SNP weighted by effect size on genetic liability to CD or UC ($PRS=\sum_{i=1}^{n}\beta_{i}\times {SNP}_{i}$, see Supplementary Table 1, Supplementary Digital Content 1, http://links.lww.com/AJG/C860). Because using merely the genome-wide significant SNP may omit some correlated informative signals that are independently associated with CD and UC, we additionally constructed genomic risk scores by including all SNP at suggestive significance level (*P* < 1 × 10^−5^) reported by the genome-wide association study. Genomic risk scores were calculated by using the LDpred2 (15). Either polygenic risk score or genomic risk score with better stratification ability was taken froward to proxy the genetic susceptibility of CD and UC and was further used to categorize the low (the lowest quintile), intermediate (quintiles 2–4), and high (highest quintile) genetic risk groups.

### Modifiable lifestyle factors

Six common lifestyle factors, including wan, were examined for their associations with CD and UC risk, respectively. These lifestyle factors were chosen based on preexisting evidence on their associations with either CD or UC, as reported by a recent umbrella review and cohort studies (11,16). Detailed information on definitions of common lifestyle factors is displayed in Supplementary Method and Supplementary Table 2 (see Supplementary Digital Content 1, http://links.lww.com/AJG/C860). Healthy lifestyle scores were constructed based on aforementioned lifestyle factors. Individuals were assigned 1 point for each of the lifestyle behaviors if they were classified into the healthy group. A higher lifestyle score indicates higher adherence to healthy lifestyle. The unweighted lifestyle score was categorized as favorable (4 or 5 healthy lifestyle factors), intermediate (3 healthy lifestyle factors), and unfavorable (0–2 healthy lifestyle factor) lifestyles. We then constructed a weighted standardized healthy lifestyle score based on the β coefficient of each lifestyle factor in the Cox proportional hazards model adjusted for age, age-square, sex, education, Townsend deprivation index, and first 20 principal components of ancestry using the formula ($\beta_{weighted}=\beta_{i}/\overset{n}{\underset{i=1}{\sum }}\beta_{i}\times 100\%$), and then, the weighted standardized healthy lifestyle score was categorized into unfavorable (the lowest quintile), intermediate (quintiles 2–4), and favorable (the highest quintile) groups.

### Cases ascertainment and follow-up

Diagnostic information was obtained from the primary care data and hospital inpatient records. Incident cases with CD and UC were ascertained by a primary or secondary diagnosis defined by corresponding *International Classification of Diseases* (*ICD*) codes (*ICD-9*: 555, 556; *ICD-10*: K50, K51). Participants were followed up from baseline (2006–2010) until the date of first diagnosis of IBD, date of death, date of loss to follow-up, the last date of hospital admission (i.e., Hospital Episode Statistics for England and Scottish Morbidity Record: March 31, 2021, and Patient Episode Database for Wales: February 28, 2018), whichever came first. Disease locations were obtained from diagnosis records for subgroup analyses (17).

### Statistical analysis

We used the Cox proportional hazards regression model to examine the associations of genetic risk categories, lifestyle categories, and genetic risk and lifestyle combined categories (9 categories with a high genetic risk and an unfavorable lifestyle as reference) with risk of incident CD and UC. The model was adjusted for age, age-square, sex, education, Townsend deprivation index, Charlson comorbidities index, and first 20 principal components of ancestry. Considering several lifestyle factors tested, we used the Bonferroni correction to account for multiple testing. The interactions between lifestyle factors and polygenic risk scores were also examined using a multiplicative interaction model. The proportionality of hazards assumption was assessed using the Schoenfeld residuals method and found to be satisfied (*P* > 0.15). To examine the consistency of the association in subpopulations, we conducted stratification analyses by age (60 years or older and younger than 60 years), sex (female and male), education attainment (≥college/university and <college/university), and the tertiles of Townsend deprivation index (from low to high, T1–T3). We also stratified the analysis on the associations of the healthy lifestyle categories with CD and UC risk by genetic risk. Sensitivity analyses and subgroup analyses by considering disease locations and age of diagnosis of UC and CD (to retrospectively include prevalent cases and stratify analysis by age of onset) were also performed to thoroughly examine their complex associations (see Supplementary Method, Supplementary Digital Content 1, http://links.lww.com/AJG/C860). The cumulative incidence of CD and UC by categories of genetic risk and lifestyle scores were obtained using the cumulative incidence function of competing risk regression (18). All tests were 2-sided, and the association with the *P* value <0.05 was deemed significant. All analyses were performed using R software, version 3.6.3.

## RESULTS

Table 1 summarizes baseline characteristics of included participants by incident disease status. Over a median follow-up of 12.0 years (interquartile range, 11.2–12.7 years), 707 cases with CD and 1,576 cases with UC were diagnosed. The median age of diagnosis was 65 years (range: 43–82 for CD) and 66 years (range: 43–82) for UC.

**Table 1.**
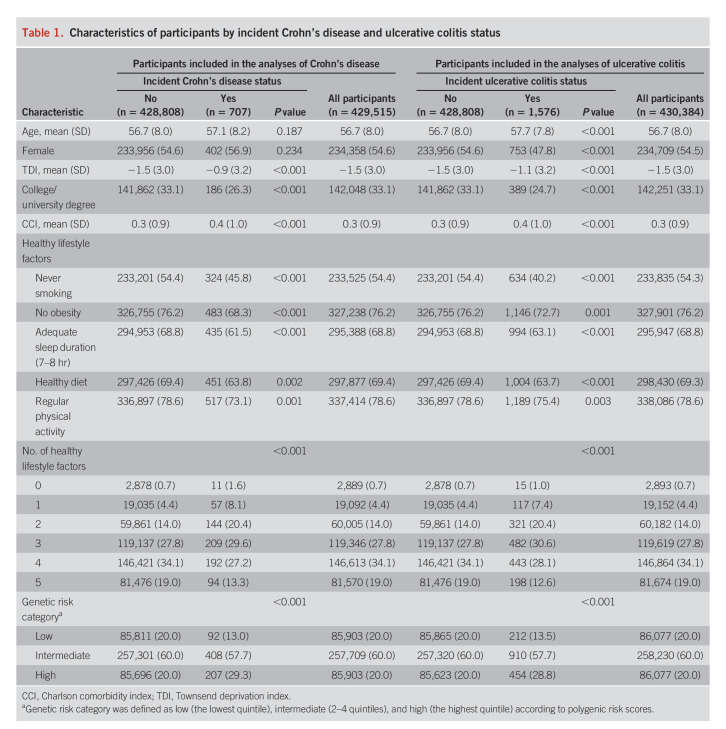
Characteristics of participants by incident Crohn's disease and ulcerative colitis status

For genetic susceptibility, both polygenic risk score and genomic risk score showed significant associations with the risk of CD and UC (see Supplementary Table 4, Supplementary Digital Content 1, http://links.lww.com/AJG/C860). Comparing with the PRS, the genomic risk score showed no further improvement on the stratification of genetic risk groups (Table 2, see Supplementary Table 4, Supplementary Digital Content 1, http://links.lww.com/AJG/C860); therefore, only the PRS was used in the joint analysis. The PRS was normally distributed (see Supplementary Figure 1, Supplementary Digital Content 1, http://links.lww.com/AJG/C860) and showed no associations with lifestyle factors with the exception for an association between polygenic risk score of CD and smoking status (see Supplementary Table 5, Supplementary Digital Content 1, http://links.lww.com/AJG/C860). Risk of incident CD and UC increased across genetic risk categories (low to high) in a linear fashion (Table 2). Compared with participants with a low genetic risk, the hazard ratios (HR) of CD and UC were 2.24 (95% confidence interval [CI] 1.75–2.86, *P* < 0.001) and 2.15 (95% CI 1.82–2.53, *P* < 0.001) for those with a high genetic risk, respectively. The associations remained significant after additional adjustment for lifestyle factors. The same pattern of associations was observed in the analysis using the genetic risk quintiles instead of categories (see Supplementary Table 4, Supplementary Digital Content 1, http://links.lww.com/AJG/C860).

**Table 2.**
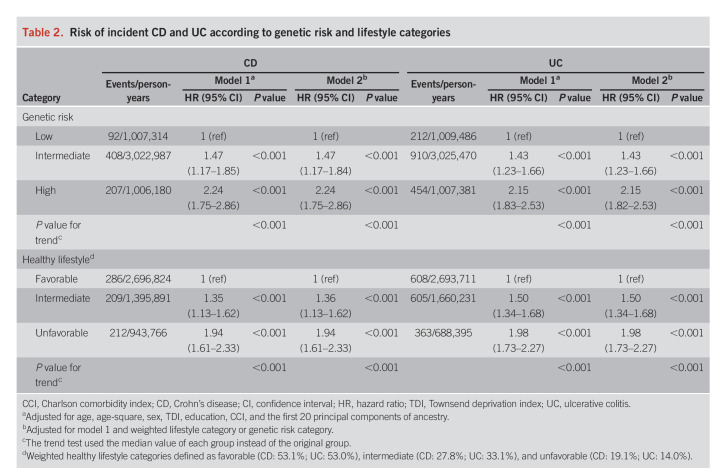
Risk of incident CD and UC according to genetic risk and lifestyle categories

The associations of individual lifestyle factors with the risk of CD and UC are summarized in Table 3. Less healthy behavior was in general associated with an increased risk of CD and UC, compared with those meeting healthy lifestyle guidelines (the reference category) for each component of healthy lifestyles, although not all risk estimates were statistically significant. Exceptions were noted for alcohol drinking, which was neither associated with CD nor associated with UC (see Supplementary Table 6, Supplementary Digital Content 1, http://links.lww.com/AJG/C860). Given that there was no well-established evidence supporting their associations from previous evidence either (12), we therefore excluded alcohol consumption from the construction of healthy lifestyle scores. For UC, the association with obesity was not statistically significant (*P* = 0.291), and irregular physical activity was marginally associated with an increased risk of UC (*P* = 0.068); nevertheless, given that obesity and physical activity were well-established lifestyle factors related to IBD based on previous evidence (19,20), we decided to include these variables for the construction of healthy lifestyle scores. In brief, both previous and current smoking were consistently associated with an increased risk of CD and UC among older populations (data not shown). We therefore simplified the smoking exposure into ever vs never smoking behaviors as one of the components of a healthier lifestyle.

**Table 3.**
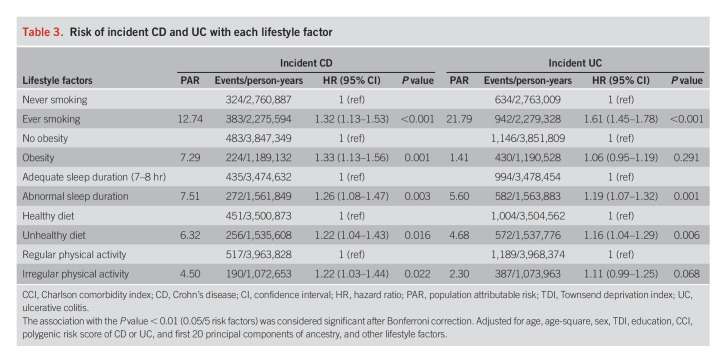
Risk of incident CD and UC with each lifestyle factor

**Figure 2.**
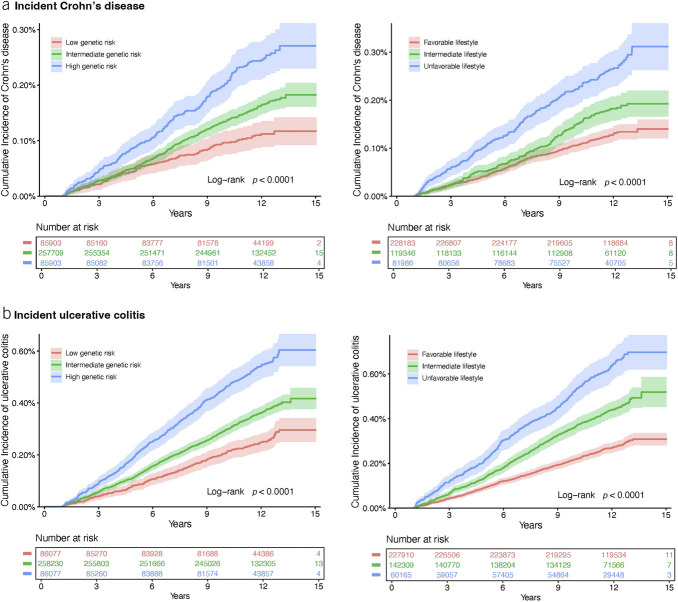
Cumulative incidence plot of the risk of incident Crohn's disease (**a**) and ulcerative colitis (**b**) by polygenic risk score categories and healthy lifestyle categories.

Having a healthier lifestyle score was significantly related to a reduced risk of CD and UC in a dose-response manner (*P* for trend < 0.001) (Table 2). The HR of CD and UC for individuals in the unfavorable category were 1.94 (95% CI 1.61–2.33; *P* < 0.001) and 1.98 (95% CI 1.73–2.27; *P* < 0.001), respectively, compared with those in the favorable category. The associations did not change in the sensitivity analysis with further adjustment for genetic risk (Table 2) in the analysis using the number of healthy lifestyle factors instead of categories (see Supplementary Table 7, Supplementary Digital Content 1, http://links.lww.com/AJG/C860) and in the analysis using the unweighted healthy lifestyle score (see Supplementary Table 8, Supplementary Digital Content 1, http://links.lww.com/AJG/C860). The cumulative incidence rate of CD and UC during the follow-up was higher in the group with an unfavorable lifestyle compared with the group with a favorable lifestyle (Figure 2).

**Figure 3.**
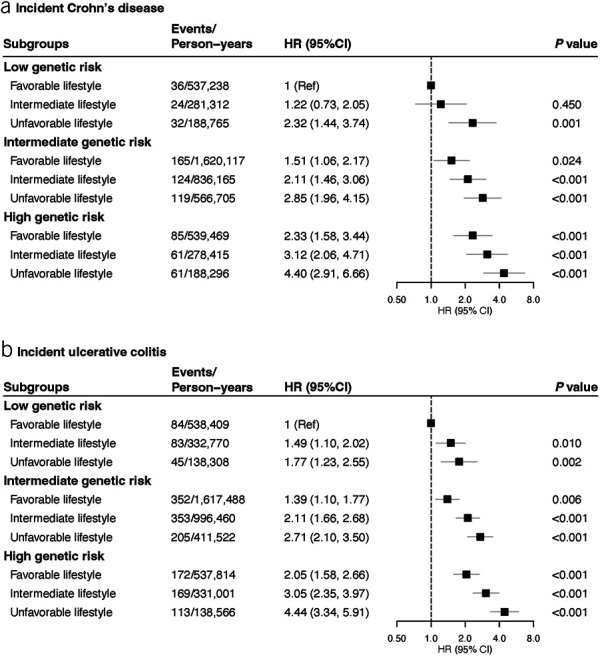
Risk of incident Crohn's disease and ulcerative colitis by joint categorization for genetic risk and healthy lifestyle score. CI, confidence interval; HR, hazard ratio.

In the analysis of joint categories for genetic risk and healthy lifestyle, the HR of CD and UC showed a linear increase with increasing genetic risk and decreasing healthy lifestyle score (Figure 3). Compared with individuals with a low genetic risk and favorable lifestyle, the HR of CD and UC for those with a high genetic risk and unfavorable lifestyle were 4.40 (95% CI 2.91–6.66; *P* < 0.001) and 4.44 (95% CI 3.34–5.91; *P* < 0.001), respectively. We observed no significant difference in the HR of CD or UC between the group of high genetic risk but having a favorable lifestyle (HR 2.33, 95% CI 1.58–3.44 for CD and HR 2.05, 95% CI 1.58–2.66 for UC) and the group of low genetic risk but having an unfavorable lifestyle (HR 2.32, 95% CI 1.44–3.74 for CD and HR 1.77, 95% CI 1.23–2.55 for UC). The analysis on the associations of healthy lifestyle categories with incident CD and UC risk in groups defined by genetic risk confirmed that the unfavorable lifestyle was associated with a higher risk of CD and UC across all genetic groups (Table 4). Specifically, in individuals with a low genetic risk, the HR of CD and UC were 2.32 (95% CI 1.42–3.81) and 1.70 (95% CI 1.17–2.47) for participants with an unfavorable lifestyle compared with those with a favorable lifestyle. We did not detect any multiplicative interaction between the genetic risk and the weighted healthy lifestyle score (*P* = 0.85 for CD and *P* = 0.87 for UC). The observed associations remained statistically significant in a series of sensitivity analyses (see Supplementary Tables 9 and 10, Supplementary Digital Content 1, http://links.lww.com/AJG/C860).

**Table 4.**
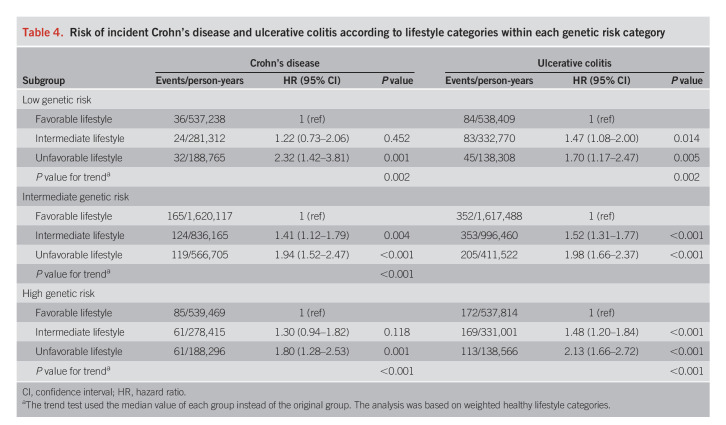
Risk of incident Crohn's disease and ulcerative colitis according to lifestyle categories within each genetic risk category

**Figure 4.**
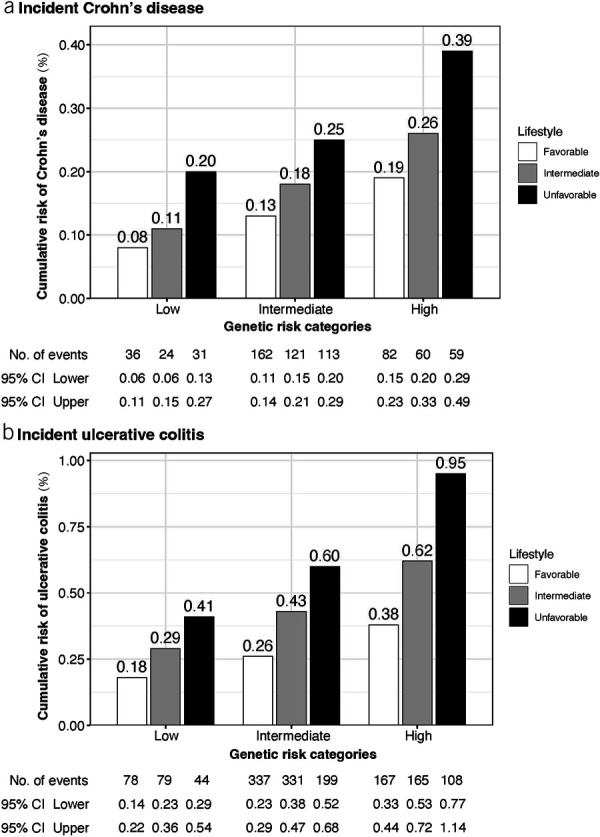
Cumulative incidence plot of the risk of incident Crohn's disease (**a**) and ulcerative colitis (**b**) by joint categorization for genetic risk and healthy lifestyle. CI, confidence interval.

We calculated the cumulative risk of CD and UC over 12 years for each group defined jointly by genetic risk and healthy lifestyle scores (Figure 4). Compared with those with a low genetic risk and favorable lifestyle (accumulative risk: 0.08% for CD, 0.18% for UC), individuals with a high genetic risk and an unfavorable lifestyle had 4.88 times higher accumulative risk of CD (equivalent to an excess risk of 0.31% due to high genetic susceptibility and an unfavorable lifestyle together) and 5.28 times higher accumulative risk of UC (equivalent to an excess risk of 0.77% due to high genetic susceptibility and unfavorable lifestyle together).

## DISCUSSION

We conducted a cohort study using data from the UK Biobank to investigate the associations across genetic susceptibility, modifiable lifestyle factors, and risk of CD and UC. We found that a polygenic risk score and modifiable lifestyle factors were independently associated with the risk of incident CD and UC. No multiplicative interaction was observed between the polygenic risk score and lifestyle scores. High genetic risk and an unfavorable lifestyle were associated with an elevated risk of CD and UC in individuals compared with their counterparts with a low genetic risk and a favorable lifestyle. The cumulative incidence of CD and UC over 12 years was approximately 5 times higher in those with the highest genetic risk and an unfavorable lifestyle compared with those with the lowest genetic risk and a favorable lifestyle.

Although no studies have been conducted to examine the effect of overall healthy lifestyle on CD or UC risk, a high adherence to a healthy lifestyle was associated with reduced mortality in patients with CD or UC from 3 large cohort studies (21). For individual lifestyle factors, there are epidemiological studies assessing the associations of each lifestyle factor with CD and UC risk. Current smoking was found to be positively associated with the risk of CD but inversely associated with risk of UC in a meta-analysis of 9 and 13 studies, respectively (22). However, most included studies are cross-sectional studies where residual confounding and reverse causality could not be eliminated. In a subsequent prospective cohort study using data from Nurses' Health Study and Nurses' Health Study II, the risk of CD was observed to increase in former and current smokers after more than 18 years of follow-up period (23). A positive association between smoking and UC risk was observed in some subsequent studies (24,25). Smoking has been also associated with the progression of CD (26), but not with that of UC (27). In this study, we performed a series of sensitivity/subgroup analyses by considering disease locations and age of diagnosis to thoroughly examine their complex associations with smoking. Our study verified that both previous or current smoking were consistently associated with an increased risk of CD regardless of disease locations and age of onset. However, the association between smoking and UC seems complex, in which smoking was associated with a reduced risk for early-onset UC (≤20 years) but an increased risk for later-onset UC (>40 years). Our previous study showed that smoking habit influences the age at diagnosis and changes in disease extent in UC (28). The mechanisms of the observed inverse association are not clear, but reverse causality is again an important point to consider. Because the initial age structure of the UK Biobank cohort is older, we therefore concentrated on older-onset cases and considered smoking as a risk factor of CD and UC.

Evidence on the association between obesity and CD is inconsistent with a positive association in a meta-analysis of 5 cohort studies (20) but a null finding in another meta-analysis (29). In a recent prospective analysis of 5 cohorts, obesity defined by body mass index (BMI) was associated with an increased risk of older-onset CD but not UC (30). A recent Mendelian randomization study found that genetically predicted higher BMI and body fat percentage was associated with an increased risk of CD, but that genetically predicted higher BMI was associated with a lower risk of UC (31). Regular physical activity has been associated with a lower risk of CD, but not UC (19,32). Although a high adherence to Mediterranean diet has been associated with a low risk of CD (33) and abnormal sleep duration has been associated with a high risk of UC (34), there are few corresponding prior studies that examined these associations jointly. Our analysis using data from the UK Biobank verified the associations of smoking, physical inactivity, unhealthy diet, and abnormal sleep duration with CD risk, with the exception of alcohol consumption. As for UC, this cohort study found positive associations between all aforementioned lifestyle factors with the risk of UC, but no significant association for alcohol drinking either. The null association of alcohol drinking with the risk of CD and UC reported by this study is consistent with evidence from a recent cohort study (12).

To our knowledge, no previous studies have examined the association of a combination of healthy lifestyle and multiple genetic factors with the risk of incident CD and UC. However, the interaction effects between genes and environmental factors, such as smoking and certain dietary nutrients, on CD and UC have been assessed. A study including 19,735 cases with IBD (10,856 cases with CD and 8,879 cases with UC) of known smoking status found that 2 variants in *HLA* and *NOD2* gene regions interacted with smoking in influencing CD risk, and smoking modified the disease risk of some variants in opposite directions for CD vs UC (35), which indicated that the effects of smoking on IBD risk depend on genetic variants. Nevertheless, an increased risk of CD and a decreased risk of UC were found in smokers in twin or sibling studies where the cases and controls shared genetic risk for the disease (36,37). The interaction effects were also observed for certain dietary nutrients, such as dietary fatty acids, potassium, and iron intake; however, these findings are far from being established to determine the gene-environment interaction on CD and UC risk (38). Our study found independent associations of genetic risk and healthy lifestyle with IBD risk, but no overall interaction for their joint effects on IBD risk. Among individuals with high genetic risk, those with an unfavorable lifestyle had double the risk of CD or UC compared with those with a favorable lifestyle. One of the interesting findings was that participants at a lower genetic risk and poor lifestyle still had an elevated risk of IBD, which implies the importance of lifestyle factors in the development of gene-less–determined IBD. However, this association was imprecise due to a low number of cases, which needs further confirmation. This finding has important clinical implications by indicating that promoting a healthy lifestyle is an effective strategy to lower incidence of these diseases, even among those with a high-risk genetic background.

Of note, age is an important factor for the associations with lifestyle factors that may have cumulative effects on IBD incidence. Given our study was based on a middle-aged and older population, our findings might not be generalized to a younger population with a shorter time exposure to poor lifestyles. Thus, the associations between lifestyle factors and IBD risk need to be re-evaluated among younger adults. In addition, future studies with frequently repeated assessments of lifestyle factors may be informative to estimate the effects of different lengths of poor lifestyle exposure time on IBD risk.

The strengths of this study include the joint analysis of the genetic and lifestyle factors to gain a comprehensive understanding on the risk of CD and UC, in which PRS and healthy lifestyle scores were constructed to examine their associations with the disease risk in a large prospective cohort of UK Biobank participants (3). We made efforts to account for additional genetic susceptibility that were not captured by genome-wide significant SNP, while the more sophisticated genomic risk score showed no superior capacity comparing with the simple PRS in risk stratification. Limitations of this study should also be acknowledged when interpreting the findings. First, adherence to a healthy lifestyle might change in the follow-up and influence the association estimation. Nonetheless, the bias caused by the change should be nondifferential and therefore attenuate the estimates in a conservative way due to the prospective nature of the design. Second, although important known confounders were adjusted in the models, there is possible residual confounding. Thus, the causality of the association for lifestyle factors cannot be exclusively determined. Third, information on lifestyle factors was collected through a self-administrative questionnaire survey, and misclassification of lifestyle factors and proneness to social desirability in responses may have occurred. Because data were collected before outcome, this misclassification is likely to be nondifferential for outcome, which typically would bias would drive any associations toward the null. In addition, misclassification of outcomes possibly caused by cases undocumented in medical records could attenuate the effect estimates. Besides, the healthy lifestyle scores have not been validated independently outside of this study due to lack of data. Fourth, the UK Biobank may not be representative of the general population due to the healthy volunteer selection bias (39). Given that the current analyses were confined to individuals of European ancestry and older population, our findings may not be generalizable to other populations of different ethnicities and/or younger age. Fifth, ICD codes were used to identify IBD conditions, which are less granular and more prone to misclassification. Although previous studies proved a good validity of ICD-defined outcomes in the UK Biobank (40,41), the observed associations for IBD that should be defined using other appropriate methods need to be warranted. Finally, our study focused only on the effects of lifestyle collected at baseline and have difficulty in detecting cumulative effects of lifestyle with age; improved lifestyle collection and repeated collection (monitoring) at intervals may allow for more detailed and accurate risk estimates of lifestyle.

In summary, both a high genetic risk and an unfavorable lifestyle were associated with an increased risk of CD and UC among adults without IBD. An unfavorable lifestyle was associated with a higher risk of CD and UC in individuals regardless of genetic strata. Adherence to a favorable lifestyle was associated with a nearly 50% lower risk of CD and UC among participants at a high genetic risk.

## CONFLICTS OF INTEREST

**Guarantor of the article:** Xue Li, PhD.

**Specific author contributions:** (CRediT Authorship Contributions) Y.S.: formal analysis: leading; methodology: leading; writing—original draft: supporting; and writing—review and editing: supporting. S.Y.: formal analysis: supporting; methodology: equal; writing—original draft: leading; and writing—review and editing: leading. X.C.: formal analysis: equal; methodology: equal; writing—original draft: supporting; and writing—review and editing: equal. J.S.: methodology: supporting; formal analysis: supporting; and writing—review and editing: supporting. R.K.: methodology: supporting; formal analysis: supporting; and writing—review and editing: supporting. L.Y.: methodology: supporting; formal analysis: supporting; and writing—review and editing: supporting: L.W.: methodology: supporting; formal analysis: supporting; and writing—review and editing: supporting. X.Z.: methodology: supporting; formal analysis: supporting; and writing—review and editing: supporting. X.K.: methodology: supporting; formal analysis: supporting; and writing—review and editing: supporting. T.K.: conceptualization: supporting; writing—original draft: supporting; and writing—review and editing: supporting. W.H.: methodology: supporting; formal analysis: supporting; and writing—review and editing: supporting. K.D.: methodology: supporting; formal analysis: supporting; and writing—review and editing: supporting. M.D.: methodology: supporting; formal analysis: supporting; and writing—review and editing: supporting. S.C.L.: conceptualization: supporting; writing—original draft: supporting; and writing—review and editing: equal. J.S.: methodology: supporting; writing—review and editing: equal. J.C.: conceptualization: leading; data curation: leading; formal analysis: equal; methodology: equal; and writing—original draft: supporting; writing—review and editing: equal. X.W.: conceptualization: equal; formal analysis: supporting; methodology: supporting; and writing—review and editing: equal. X.L.: conceptualization: leading; data curation: leading; formal analysis: equal; methodology: equal; and writing—original draft: equal; writing—review and editing: equal. E.T.: conceptualization: equal; methodology: equal; writing—review and editing: equal. E.L.G.: conceptualization: equal; methodology: equal; and writing—review and editing: equal. All authors critically reviewed the manuscript for important intellectual content. The corresponding author attests that all listed authors meet authorship criteria and that no others meeting the criteria have been omitted.

**Financial support:** X.L.: the Natural Science Fund for Distinguished Young Scholars of Zhejiang Province (LR22H260001). X.Y.W.: National Natural Science Foundation of China (81970494) and Key Project of Research and Development Plan of Hunan Province(2019SK2041). S.C.L.: the Swedish Heart-Lung Foundation (Hjärt-Lungfonden, 20210351), the Swedish Research Council (Vetenskapsrådet, 2019-00977), and the Swedish Cancer Society (Cancerfonden). E.T.: CRUK Career Development Fellowship (C31250/A22804). K.F.D.: Project of the regional diagnosis and treatment center of the Health Planning Committee (No. JBZX-201903).

**Potential competing interests:** The authors declare no competing interests.

**Ethical approval:** This study was covered by the ethical approval for the UK Biobank studies from the North West Multi-centre Research Ethics Committee (MREC), and written informed consent was obtained from all participants.

**Data sharing:** Researchers can request the data we used from the UK Biobank (www.ukbiobank.ac.uk/).

**Transparency:** The lead author (X.L.) affirms that the manuscript is an honest, accurate, and transparent account of the study being reported; that no important aspects of the study have been omitted; and that any discrepancies from the study as planned have been explained.

**Dissemination to participants and related patient and public communities:** The results of the research will be disseminated to the public through broadcasts, popular science articles, and newspapers.

## Supplementary Material

SUPPLEMENTARY MATERIAL

## Notes

Study Highlights WHAT IS KNOWN

✓ Previous studies have identified many genetic variants and several modifiable risk factors for inflammatory bowel disease (IBD).
✓ Data are limited on the combined effects of genetic and lifestyle factors on IBD risk.

WHAT IS NEW HERE

✓ Genetic risk and modifiable lifestyle factors were independently associated with the risk of incident Crohn's disease and ulcerative colitis.
✓ Adherence to a favorable lifestyle was associated with a nearly 50% lower risk of Crohn's disease and ulcerative colitis among participants at a high genetic risk.
