# Supplementary material for: The Contribution of Genetic Risk and Lifestyle Factors in the Development of Adult-Onset Inflammatory Bowel Disease: A Prospective Cohort Study
Source: Am J Gastroenterol. 2023 Jan 9;118(3):511–22. doi: 10.14309/ajg.0000000000002180 (PMC9973435; doi:10.14309/ajg.0000000000002180)
Supplement: SUPPLEMENTARY MATERIAL [file acg-118-511-s001.docx]

Supporting information for

**The contribution of genetic risk and lifestyle factors in the development of adult-onset inflammatory bowel disease: a prospective cohort study**

*Yuhao Sun, Shuai Yuan, Xuejie Chen, Jing Sun, Rahul Kalla, Lili Yu, Lijuan Wang, Xuan Zhou, Xiangxing Kong, Therese Hesketh, Gwo-tzer Ho, Kefeng Ding, Malcolm Dunlop, Susanna C. Larsson, Jack Satsangi, Jie Chen, Xiaoyan Wang, Xue Li, Evropi Theodoratou, Edward L Giovannucci*

**Supplementary Methods**

**Supplementary Tables and Figures**

**Supplementary Table 1.** Genetic variants associated with Crohn’s disease and ulcerative colitis

**Supplementary Table 2.** Definitions of lifestyle factors

**Supplementary Table 3.** Definitions of major depressive disorder

**Supplementary Table 4.** Risk of incident Crohn's disease and ulcerative colitis according to polygenic risk score and genomic risk score

**Supplementary Table 5.** Associations between the polygenic risk score of Crohn’s disease, ulcerative colitis and lifestyle factors

**Supplementary Table 6.** Risk of incident Crohn's disease and ulcerative colitis according to alcohol consumption

**Supplementary Table 7**. Risk of incident Crohn's disease and ulcerative colitis by number of healthy lifestyle factors

**Supplementary Table 8**. Risk of incident Crohn's disease and ulcerative colitis by unweighted healthy lifestyle categories

**Supplementary Table 9**. Risk of Incident Crohn’s disease by genetic and lifestyle risk after excluding incident Crohn’s disease within 2 years after baseline, after excluding baseline colorectal cancer, additionally adjusted for depression, with unweighted lifestyle score, weighted lifestyle score in multivariate imputed data, and after excluding participants with incomplete data of covariables

**Supplementary Table 10**. Risk of Incident ulcerative colitis by genetic and lifestyle risk after excluding incident Crohn’s disease within 2 years after baseline, after excluding baseline colorectal cancer, additionally adjusted for depression, with unweighted lifestyle score, weighted lifestyle score in multivariate imputed data, and after excluding participants with incomplete data of covariables

**Supplementary Figure 1**. Distribution of the polygenic risk score for Crohn’s disease (A) and ulcerative colitis (B)

**Supplementary Methods**

**Lifestyle factors**

Specifically, smoking status was defined as never or ever smoking, and smoking frequency (only occasionally, most or all day) of previous and current smokers was also considered in further subgroup analyses. BMI was categorized as non-obese (<30.0 kg/m^2^) and obese (≥30.0 kg/m^2^) according to World Health Organization standards for the European population. Sleep duration was defined as normal (7.0-8.0 hours/day) and abnormal sleep duration (<7 or >8 hours/day).^1^ A dietary pattern including 7 common foods was used to assess the diet quality according to the American dietary guidelines.^2, 3^ Dietary intake information was collected by a food frequency questionnaire. One diet point was given if the intakes were met for a) fruits ≥3 times/day; b) vegetables ≥3 times/day; c) fish ≥2 times/week; d) whole grains ≥3 times/day; e) refined grains ≤1.5 times/day; f) processed meats ≤1 times/week; and g) unprocessed red meats ≤2.5 times/week. The diet score ranged from 0 to 7, and a diet score ≥4 indicated a high adherence to a healthy dietary pattern. Healthy alcohol drinking was defined as never to light alcohol consumption (0 to 14 g/d for women and 0 to 28 g/d for men) with the maximum limit reflecting the American dietary guidelines. Regular physical activity was defined by at least ≥150 minutes moderate activity per week or ≥75 minutes vigorous activity per week (or an equivalent combination) or having moderate physical activity at least 5 days a week or vigorous activity once a week, as recommended by the American Heart Association.^4^

**Sensitivity analysis**

The associations were further examined with several sensitivity analyses, including: 1) an analysis using the genetic risk quintiles instead of categories; 2) an analysis using the number of healthy lifestyle factors instead of categories; 3) an analysis excluding incident CD or UC within 2 years after baseline; 4) an analysis excluding baseline colorectal cancer patients; 5) an analysis using an unweighted lifestyle score; 6) an analysis with further adjustment for baseline depressive symptoms; 7) an analysis using multivariate imputed covariate data; 8) an analysis excluding participants with incomplete covariate data.

**References**

1. Ananthakrishnan AN, Khalili H, Konijeti GG, et al. Sleep duration affects risk for ulcerative colitis: a prospective cohort study. Clin Gastroenterol Hepatol 2014;12:1879-86.

2. Mozaffarian D, Appel LJ, Van Horn L. Components of a cardioprotective diet: new insights. Circulation 2011;123:2870-91.

3. US Department of Health and Human Services. 2015-2020 Dietary guidelines for Americans. 8th edition. December 2015.

4. Lloyd-Jones DM, Hong Y, Labarthe D, et al. Defining and setting national goals for cardiovascular health promotion and disease reduction: the American Heart Association's strategic Impact Goal through 2020 and beyond. Circulation 2010;121:586-613.

Supplementary Table 1. Genetic variants associated with Crohn’s disease and ulcerative colitis

| **Outcome** | **SNP** | **Chr** | **Pos_hg19** | **EA** | **NEA** | **Beta** | **SE** | ***P* value** |
| --- | --- | --- | --- | --- | --- | --- | --- | --- |
| CD | rs7517847 | 1 | 67681669 | C | A | -0.342 | 0.017 | 2.29×10^-46 |
| CD | rs7517810 | 1 | 172853460 | A | G | 0.131 | 0.012 | 1.11×10^-14 |
| CD | rs3024505 | 1 | 206939904 | A | G | 0.166 | 0.014 | 3.91×10^-09 |
| CD | rs10798069 | 1 | 186875459 | A | C | -0.073 | 0.011 | 4.25×10^-09 |
| CD | rs7555082 | 1 | 198598663 | A | G | 0.122 | 0.018 | 1.47×10^-10 |
| CD | rs10495903 | 2 | 43806918 | A | G | 0.122 | 0.015 | 3.3×10^-08 |
| CD | rs6708413 | 2 | 103063369 | G | A | 0.113 | 0.013 | 1.54×10^-10 |
| CD | rs6716753 | 2 | 231097129 | G | A | 0.131 | 0.013 | 1.87×10^-08 |
| CD | rs12994997 | 2 | 234173503 | G | A | -0.223 | 0.015 | 2.06×10^-37 |
| CD | rs11681525 | 2 | 145492382 | C | G | -0.151 | 0.024 | 4.08×10^-11 |
| CD | rs35320439 | 2 | 242737341 | G | A | 0.086 | 0.009 | 9.89×10^-10 |
| CD | rs3197999 | 3 | 49721532 | A | G | 0.157 | 0.011 | 9.13×10^-13 |
| CD | rs11742570 | 5 | 40410584 | A | G | -0.248 | 0.016 | 4.47×10^-34 |
| CD | rs1363907 | 5 | 96252803 | A | G | 0.104 | 0.012 | 1.46×10^-11 |
| CD | rs11743851 | 5 | 130613600 | G | A | 0.140 | 0.011 | 8.69×10^-12 |
| CD | rs11741861 | 5 | 150277909 | G | A | 0.285 | 0.015 | 4.6×10^-16 |
| CD | rs6556412 | 5 | 158787385 | A | G | 0.157 | 0.011 | 8.03×10^-15 |
| CD | rs6908425 | 6 | 20728731 | A | G | -0.105 | 0.017 | 2.42×10^-08 |
| CD | rs7746082 | 6 | 106435269 | C | G | 0.131 | 0.012 | 1.77×10^-08 |
| CD | rs1819333 | 6 | 167373547 | C | A | -0.117 | 0.014 | 9.3×10^-12 |
| CD | rs7773324 | 6 | 382559 | G | A | -0.083 | 0.011 | 1.06×10^-09 |
| CD | rs13204048 | 6 | 3420406 | G | A | -0.073 | 0.011 | 2.89×10^-08 |
| CD | rs7758080 | 6 | 149577079 | G | A | 0.077 | 0.009 | 7.27×10^-09 |
| CD | rs1456896 | 7 | 50304461 | G | A | -0.094 | 0.014 | 2.9×10^-08 |
| CD | rs921720 | 8 | 126534671 | A | G | -0.117 | 0.014 | 6.4×10^-12 |
| CD | rs4246905 | 9 | 117553249 | A | G | -0.139 | 0.016 | 1.28×10^-14 |
| CD | rs10781499 | 9 | 139266405 | A | G | 0.166 | 0.010 | 1.03×10^-19 |
| CD | rs11010067 | 10 | 35295431 | G | C | 0.131 | 0.011 | 2.33×10^-09 |
| CD | rs10761659 | 10 | 64445564 | A | G | -0.186 | 0.015 | 3.42×10^-19 |
| CD | rs4409764 | 10 | 101284237 | A | C | 0.174 | 0.010 | 8.48×10^-19 |
| CD | rs2155219 | 11 | 76299194 | A | C | 0.174 | 0.010 | 6.51×10^-13 |
| CD | rs12422544 | 12 | 40528432 | G | A | 0.378 | 0.025 | 3.29×10^-12 |
| CD | rs7954567 | 12 | 6491125 | A | G | 0.086 | 0.009 | 1.3×10^-09 |
| CD | rs3764147 | 13 | 44457925 | G | A | 0.140 | 0.012 | 7.31×10^-09 |
| CD | rs9525625 | 13 | 43018030 | A | G | 0.077 | 0.009 | 1.41×10^-09 |
| CD | rs17293632 | 15 | 67442596 | A | G | 0.131 | 0.012 | 1.08×10^-12 |
| CD | rs26528 | 16 | 28517709 | G | A | 0.122 | 0.011 | 1.06×10^-08 |
| CD | rs3091315 | 17 | 32593665 | G | A | -0.139 | 0.016 | 9.52×10^-12 |
| CD | rs12946510 | 17 | 37912377 | A | G | 0.122 | 0.011 | 4.3×10^-08 |
| CD | rs3853824 | 17 | 54880993 | A | G | -0.083 | 0.011 | 1.17×10^-10 |
| CD | rs1893217 | 18 | 12809340 | G | A | 0.166 | 0.014 | 1.92×10^-12 |
| CD | rs7236492 | 18 | 77220616 | A | G | -0.094 | 0.022 | 9.09×10^-09 |
| CD | rs2024092 | 19 | 1124031 | A | G | 0.148 | 0.012 | 2.26×10^-11 |
| CD | rs11879191 | 19 | 10512911 | A | G | -0.139 | 0.019 | 1.66×10^-08 |
| CD | rs516246 | 19 | 49206172 | A | G | 0.113 | 0.011 | 1.21×10^-08 |
| CD | rs6062504 | 20 | 62348907 | A | G | -0.105 | 0.015 | 3.28×10^-10 |
| CD | rs2823286 | 21 | 16817938 | A | G | -0.139 | 0.015 | 1.24×10^-09 |
| CD | rs7282490 | 21 | 45615741 | G | A | 0.122 | 0.011 | 3.81×10^-13 |
| CD | rs2256609 | 22 | 21925017 | G | A | 0.104 | 0.014 | 8.02×10^-09 |
| CD | rs2413583 | 22 | 39659773 | A | G | -0.211 | 0.021 | 3.65×10^-10 |
| CD | rs727563 | 22 | 41867377 | G | A | 0.095 | 0.009 | 1.88×10^-10 |
| UC | rs6667605 | 1 | 2502780 | A | G | -0.083 | 0.014 | 1.4×10^-08 |
| UC | rs3806308 | 1 | 20142866 | A | G | -0.174 | 0.016 | 9.81×10^-15 |
| UC | rs6426833 | 1 | 20171860 | G | A | -0.236 | 0.017 | 4.86×10^-31 |
| UC | rs12568930 | 1 | 22702231 | G | A | -0.128 | 0.021 | 6.24×10^-11 |
| UC | rs7517847 | 1 | 67681669 | C | A | -0.151 | 0.015 | 6.03×10^-11 |
| UC | rs1801274 | 1 | 161479745 | G | A | -0.174 | 0.016 | 3.78×10^-17 |
| UC | rs7554511 | 1 | 200877562 | A | C | -0.163 | 0.017 | 1.05×10^-11 |
| UC | rs3024505 | 1 | 206939904 | A | G | 0.223 | 0.013 | 2.97×10^-17 |
| UC | rs7608910 | 2 | 61204856 | G | A | 0.131 | 0.012 | 1.81×10^-12 |
| UC | rs3749171 | 2 | 241569692 | A | G | 0.140 | 0.014 | 2.33×10^-10 |
| UC | rs9868809 | 3 | 48681053 | A | G | 0.148 | 0.018 | 6.01×10^-09 |
| UC | rs113010081 | 3 | 46457412 | G | A | 0.131 | 0.018 | 9.02×10^-10 |
| UC | rs2189234 | 4 | 106075498 | A | C | 0.077 | 0.009 | 1.95×10^-10 |
| UC | rs254560 | 5 | 134443606 | A | G | 0.077 | 0.012 | 1.6×10^-08 |
| UC | rs56167332 | 5 | 158827769 | A | C | 0.140 | 0.011 | 5.3×10^-11 |
| UC | rs6920220 | 6 | 138006504 | A | G | 0.148 | 0.013 | 5.22×10^-11 |
| UC | rs7805114 | 7 | 107450033 | C | A | -0.117 | 0.015 | 5.86×10^-09 |
| UC | rs1077773 | 7 | 17442679 | G | A | -0.073 | 0.011 | 5.96×10^-09 |
| UC | rs4246905 | 9 | 117553249 | A | G | -0.117 | 0.016 | 1.97×10^-08 |
| UC | rs10781499 | 9 | 139266405 | A | G | 0.131 | 0.012 | 7.69×10^-13 |
| UC | rs4409764 | 10 | 101284237 | A | C | 0.157 | 0.011 | 8.39×10^-15 |
| UC | rs2155219 | 11 | 76299194 | A | C | 0.122 | 0.012 | 1.48×10^-09 |
| UC | rs483905 | 11 | 96023427 | A | G | 0.086 | 0.013 | 1.57×10^-08 |
| UC | rs561722 | 11 | 114386830 | A | G | -0.128 | 0.016 | 5.21×10^-09 |
| UC | rs7134472 | 12 | 68499986 | A | G | 0.157 | 0.011 | 4.77×10^-18 |
| UC | rs17085007 | 13 | 27531267 | G | A | 0.131 | 0.014 | 1.18×10^-08 |
| UC | rs12946510 | 17 | 37912377 | A | G | 0.131 | 0.012 | 4.95×10^-10 |
| UC | rs17736589 | 17 | 76737118 | G | A | 0.086 | 0.019 | 4.34×10^-08 |
| UC | rs2836878 | 21 | 40465534 | A | G | -0.223 | 0.019 | 2.05×10^-20 |
| UC | rs7282490 | 21 | 45615741 | G | A | 0.104 | 0.012 | 7.08×10^-11 |

CD, Crohn's disease; Chr, chromosome; EA, effect allele; NEA, non-effect allele; SNP; single nucleotide polymorphism; UC, ulcerative colitis.

**l**

Supplementary Table 2. Definitions of lifestyle factors

| **Lifestyle factors** | **Definitions** | **UK Biobank field code** |
| --- | --- | --- |
| **Smoking** | 1-Ever smoking (both previous and current)  UK Biobank Touchscreen questionnaire at baseline divided the smoking status into three categories: never, previous and current | 20116 |
| **BMI** | 1-BMI ≥18.5 and <25.0  UK Biobank measured body composition manually at baseline, and BMI was constructed from height and weight measured (weight/height-square, Kg/m2) | 21001 |
| **Sleep** | 1-Sleep duration ≥7 and ≤8  UK Biobank Touchscreen question 'About how many hours sleep do you get in every 24 hours? (Please include naps)' | 1160 |
| **Diet** | 1-Consuming 4-7 adequate amounts of the recommended food groups  UK Biobank Food Frequency Questionnaire asked the frequency of intake of a range of common food and drink items.  7 recommended food groups and adequate amounts: 1. Fruits: ≥ 3 times/day 2. Vegetables: ≥ 3 times/day 3. Fish: ≥2 times/week 4. Whole grains: ≥ 3 times/day 5. Refined grains: ≤1.5 times/day 6. Processed meats: ≤ 1 times/week 7. Unprocessed red meats: ≤ 2.5 times/week | 1309, 1319, 1289, 1299, 1329, 1339, 1349, 1369, 1379, 1389, 1438, 1448, 1458, 1468 |
| **Physical activity** | 1-regular physical activity  UK Biobank Touchscreen questionnaire on the reported type and duration of physical activity (including walking, DIY, moderate and vigorous physical activity, strenuous sports, etc).  One of the following is considered as regular physical activity:  1.≥150 minutes moderate activity per week  2.≥ 75 minutes vigorous activity per week  3. Equivalent combination  4. Moderate physical activity at least 5 days a week or vigorous activity once a week | 884, 894, 904, 914 |
| **Alcohol consumption** | 1-Moderate consumption (women: >0 and ≤14g/day; men: >0 and ≤28g/day)  UK Biobank Touchscreen questionnaire on whether the participant reported drinking alcohol, frequency of intake, beverage type, whether they usually drink with meals.  According to the US Dietary Guidelines for Americans 2015-2020, up to 1 drink/day for women and up to 2 drinks/day for men. To calculate drink-equivalents as per guidelines, multiply the volume in ounces by the alcohol content in percent and divide by 0.6 ounces of alcohol per drink-equivalent; then convert to grams: 1 drink-equivalent described as containing 14g of pure alcohol.  125ml wine=0.85 drink-equivalents,  4% ABV pint beer = 1.28 drink-equivalents,  25ml spirits=0.57 drink-equivalents,  50ml fortified wine= 0.56 drink-equivalents | 1558, 1568, 1578, 1588, 1598, 1608, 5364, 4407, 4418, 4429, 4440, 4451, 4462 |

Supplementary Table 3. Definitions of major depressive disorder

| **ACE touchscreen question** | **Depression symptom (with score >= 3):** | **UKB Code** |
| --- | --- | --- |
| Over the past two weeks, how often have you felt down, depressed or hopeless? | 0 (not at all); 1 (several days); 2 (more than half the days); 3 (nearly every day) | 2050 |
| Over the past two weeks, how often have you had little interest or pleasure in doing things? | 0 (not at all); 1 (several days); 2 (more than half the days); 3 (nearly every day) | 2060 |

Supplementary Table 4. Risk of incident Crohn's disease and ulcerative colitis according to polygenic risk score and genomic risk score

|  | **Crohn's disease** | | | | | **Ulcerative colitis** | | | | |
| --- | --- | --- | --- | --- | --- | --- | --- | --- | --- | --- |
| **Category** | **Events/**  **Person-years** | **Model 1 ^a^** |  | **Model 2 ^b^** |  | **Events/Person-years** | **Model 1 ^a^** |  | **Model 2 ^b^** |  |
|  |  | **HR (95% CI)** | ***P* value** | **HR (95% CI)** | ***P* value** |  | **HR (95% CI)** | ***P* value** | **HR (95% CI)** | ***P* value** |
| Low | 115/1,006,604 | 1 (Ref) |  | 1 (Ref) |  | 230/1,008,599 | 1 (Ref) |  | 1 (Ref) |  |
| Intermediate | 394/3,022,021 | 1.13 (0.92, 1.40) | 0.235 | 1.14 (0.92, 1.40) | 0.229 | 908/3,025,318 | 1.32 (1.14, 1.52) | <0.001 | 1.92 (1.64, 2.25) | <0.001 |
| High | 198/1,007,856 | 1.70 (1.35, 2.14) | <0.001 | 1.70 (1.35, 2.14) | <0.001 | 438/1,008,420 | 1.92 (1.63, 2.25) | <0.001 | 1.92 (1.64, 2.25) | <0.001 |
| *p* value for trend ^c^ |  |  | <0.001 |  | <0.001 |  |  | <0.001 |  | <0.001 |
| 1 (the lowest) | 115/1,006,604 | 1 (Ref) |  | 1 (Ref) |  | 230/1,008,599 | 1 (Ref) |  | 1 (Ref) |  |
| 2 | 109/1,007,185 | 0.94 (0.73, 1.23) | 0.666 | 0.95 (0.73, 1.23) | 0.672 | 251/1,008,132 | 1.09 (0.91, 1.30) | 0.344 | 1.09 (0.91, 1.30) | 0.342 |
| 3 | 136/1,008,247 | 1.17 (0.92, 1.51) | 0.205 | 1.18 (0.92, 1.51) | 0.197 | 299/1,008,432 | 1.30 (1.10, 1.54) | 0.003 | 1.30 (1.10, 1.54) | 0.003 |
| 4 | 149/1,006,589 | 1.29 (1.01, 1.64) | 0.044 | 1.29 (1.01, 1.64) | 0.042 | 358/1,008,754 | 1.57 (1.33, 1.85) | <0.001 | 1.57 (1.33, 1.85) | <0.001 |
| 5 (the highest) | 198/1,007,856 | 1.70 (1.35, 2.14) | <0.001 | 1.70 (1.35, 2.15) | <0.001 | 438/1,008,420 | 1.92 (1.63, 2.25) | <0.001 | 1.92 (1.64, 2.25) | <0.001 |
| *p* value for trend ^c^ |  |  | <0.001 |  | <0.001 |  |  | <0.001 |  | <0.001 |
| 1 (the lowest) | 92/1,007,314 | 1 (Ref) |  | 1 (Ref) |  | 212/1,009,486 | 1 (Ref) |  | 1 (Ref) |  |
| 2 | 115/1,007,410 | 1.25 (0.95, 1.64) | 0.113 | 1.25 (0.95, 1.64) | 0.114 | 246/1,008,916 | 1.16 (0.97, 1.39) | 0.112 | 1.16 (0.97, 1.40) | 0.108 |
| 3 | 135/1,008,087 | 1.46 (1.12, 1.90) | 0.005 | 1.46 (1.12, 1.90) | 0.005 | 322/1,008,795 | 1.52 (1.28, 1.81) | <0.001 | 1.52 (1.28, 1.81) | <0.001 |
| 4 | 158/1,007,491 | 1.71 (1.32, 2.21) | <0.001 | 1.71 (1.32, 2.21) | <0.001 | 342/1,007,759 | 1.62 (1.36, 1.92) | <0.001 | 1.62 (1.36, 1.92) | <0.001 |
| 5 (the highest) | 207/1,006,180 | 2.24 (1.75, 2.86) | <0.001 | 2.24 (1.75, 2.86) | <0.001 | 454/1,007,381 | 2.15 (1.83, 2.53) | <0.001 | 2.15 (1.82, 2.53) | <0.001 |
| *p* value for trend ^c^ |  |  | <0.001 |  | <0.001 |  |  | <0.001 |  | <0.001 |

CI indicates confidence interval; HR, hazard ratio.

^a^ Adjusted for age, age-square, sex, TDI, education, CCI, and first 20 principal components of ancestry.

^b^ Adjusted for Model 1 and weighted lifestyle categories

^c^ The trend test used the median value of each group instead of the original group.

Supplementary Table 5. Associations between the polygenic risk score of Crohn’s disease, ulcerative colitis and lifestyle factors

| **Lifestyle Factors** | **Polygenic risk score of CD** | | **Polygenic risk score of UC** | |
| --- | --- | --- | --- | --- |
|  | **OR (95% CI)** | ***P* value** | **OR (95% CI)** | ***P* value** |
| Never smoking | 1.01 (1.01, 1.02) | 0.002 | 1.00 (0.99, 1.01) | 0.910 |
| No obesity | 1.00 (0.99, 1.02) | 0.442 | 1.00 (0.99, 1.02) | 0.724 |
| Adequate sleep duration (7-8h) | 1.00 (0.99, 1.01) | 0.670 | 0.99 (0.98, 1.01) | 0.350 |
| Healthy diet | 0.99 (0.98, 1.00) | 0.137 | 0.99 (0.98, 1.01) | 0.324 |
| Regular physical activity | 0.99 (0.98, 1.00) | 0.212 | 1.00 (0.99, 1.02) | 0.536 |

CD, Crohn's disease; UC, ulcerative colitis; CI indicates confidence interval; OR, odds ratio.

Adjusted for age, age-square, sex, TDI, education, CCI, and first 20 principal components of ancestry, and other healthy lifestyle factors.

Supplementary Table 6. Risk of incident Crohn's disease and ulcerative colitis according to alcohol consumption

| **Alcohol consumption** | **Crohn's disease (total n=367,703, cases n=579)** | | | | | **Ulcerative colitis (total n=368,474, cases n=1350)** | | | | |
| --- | --- | --- | --- | --- | --- | --- | --- | --- | --- | --- |
|  | **Events/**  **Person-years** | **Model 1 ^a^** |  | **Model 2 ^b^** |  | **Events/Person-years** | **Model 1 ^a^** |  | **Model 2 ^b^** |  |
|  |  | **HR (95% CI)** | ***P* value** | **HR (95% CI)** | ***P* value** |  | **HR (95% CI)** | ***P* value** | **HR (95% CI)** | ***P* value** |
| Moderate | 325/2,496,909 | 1 (Ref) |  | 1 (Ref) |  | 746/2,499,793 | 1 (Ref) |  | 1 (Ref) |  |
| Inappropriate | 254/1,791,212 | 0.99 (0.84, 1.18) | 0.946 | 0.99 (0.84, 1.18) | 0.948 | 604/1,793,548 | 1.03 (0.92, 1.15) | 0.632 | 1.03 (0.92, 1.15) | 0.632 |

CI indicates confidence interval; HR, hazard ratio.

^a^ Adjusted for age, age-square, sex, TDI, education, CCI, and the first 20 principal components of ancestry; and other lifestyle factors in the analysis of alcohol consumption.

^b^ Adjusted for Model 1 and genetic risk category.

Supplementary Table 7. Risk of incident Crohn's disease and ulcerative colitis by number of healthy lifestyle factors

| **Number of healthy lifestyle factors** | **Events/Person-years** | **Model 1 ^a^** | | **Model 2 ^b^** | |
| --- | --- | --- | --- | --- | --- |
|  |  | **HR (95% CI)** | ***P* value** | **HR (95% CI)** | ***P* value** |
| Crohn's disease |  |  |  |  |  |
| 5 | 94/967,897 | 1 (Ref) |  | 1 (Ref) |  |
| 4 | 192/1,728,927 | 1.11 (0.87, 1.42) | 0.402 | 1.11 (0.87, 1.42) | 0.402 |
| 3 | 209/1,395,891 | 1.45 (1.14, 1.86) | 0.003 | 1.46 (1.14, 1.86) | 0.003 |
| 2 | 144/693,449 | 1.95 (1.50, 2.54) | <0.001 | 1.95 (1.50, 2.55) | <0.001 |
| 1 | 57/217,805 | 2.37 (1.69, 3.32) | <0.001 | 2.37 (1.69, 3.33) | <0.001 |
| 0 | 11/32,512 | 3.00 (1.60, 5.64) | 0.001 | 3.02 (1.61, 5.67) | 0.001 |
| *p* value for trend ^c^ |  |  | <0.001 |  | <0.001 |
| Ulcerative colitis |  |  |  |  |  |
| 5 | 198/968,658 |  |  |  |  |
| 4 | 443/1,730,576 | 1.18 (1.00, 1.40) | 0.051 | 1.18 (1.00, 1.40) | 0.052 |
| 3 | 482/1,397,726 | 1.50 (1.27, 1.78) | <0.001 | 1.50 (1.27, 1.78) | <0.001 |
| 2 | 321/694,616 | 1.90 (1.59, 2.28) | <0.001 | 1.90 (1.59, 2.28) | <0.001 |
| 1 | 117/218,201 | 2.11 (1.67, 2.66) | <0.001 | 2.10 (1.66, 2.65) | <0.001 |
| 0 | 15/32,559 | 1.75 (1.03, 2.96) | 0.039 | 1.74 (1.03, 2.95) | 0.040 |
| *p* value for trend ^c^ |  |  | <0.001 |  | <0.001 |

CI indicates confidence interval; HR, hazard ratio.

^a^ Adjusted for age, age-square, sex, TDI, education, CCI, and first 20 principal components of ancestry.

^b^ Adjusted for Model 1 and genetic risk categories

^c^ The trend test used the median value of each group instead of the original group.

Supplementary Table 8. Risk of incident Crohn's disease and ulcerative colitis by unweighted healthy lifestyle categories

| **Unweighted healthy lifestyle categories** | **Events/Person-years** | **Model 1 ^a^** | | **Model 2 ^b^** | |
| --- | --- | --- | --- | --- | --- |
|  |  | **HR (95% CI)** | ***P* value** | **HR (95% CI)** | ***P* value** |
| Crohn's disease |  |  |  |  |  |
| Favorable | 290/2,679,393 | 1 (Ref) |  | 1 (Ref) |  |
| Intermediate | 206/1,333,305 | 1.35 (1.13, 1.62) | <0.001 | 1.36 (1.13, 1.62) | <0.001 |
| Unfavorable | 201/875,841 | 1.94 (1.61, 2.33) | <0.001 | 1.94 (1.61, 2.33) | <0.001 |
| *p* value for trend ^c^ |  |  | <0.001 |  | <0.001 |
| Ulcerative colitis |  |  |  |  |  |
| Favorable | 254/1,176,187 | 1 (Ref) |  | 1 (Ref) |  |
| Intermediate | 513/1,925,357 | 1.34 (1.19, 1.51) | <0.001 | 1.34 (1.19, 1.51) | <0.001 |
| Unfavorable | 755/1,806,103 | 1.73 (1.53, 1.96) | <0.001 | 1.73 (1.53, 1.96) | <0.001 |
| *p* value for trend ^c^ |  |  | <0.001 |  | <0.001 |

CI indicates confidence interval; HR, hazard ratio.

^a^ Adjusted for age, age-square, sex, TDI, education, CCI, and first 20 principal components of ancestry.

^b^ Adjusted for Model 1 and genetic risk categories.

^c^ The trend test used the median value of each group instead of the original group.

Supplementary Table 9. Risk of Incident Crohn’s disease by genetic and lifestyle risk after excluding incident Crohn’s disease within 2 years after baseline, after excluding baseline colorectal cancer, additionally adjusted for depression, with unweighted lifestyle score, weighted lifestyle score in multivariate imputed data, and after excluding participants with incomplete data of covariables

| **Subgroup** | **Incident CD within 2 years after baseline excluded (n=429,441)** | | | **Prevalent colorectal cancer at baseline excluded (n=427,432)** | | | **Additionally adjusted for depression (n=429,515)** | | |
| --- | --- | --- | --- | --- | --- | --- | --- | --- | --- |
|  | **Events/Person-years** | **HR (95% CI)** | ***P* value** | **Events/Person-years** | **HR (95% CI)** | ***P* value** | **Events/Person-years** | **HR (95% CI)** | ***P* value** |
| Low genetic risk | |  |  |  |  |  |  |  |  |
| Favorable lifestyle | 31/537,230 | 1 (Ref) |  | 35/534,975 | 1 (Ref) |  | 36/537,238 | 1 (Ref) |  |
| Intermediate lifestyle | 22/281,309 | 1.30 (0.75, 2.25) | 0.341 | 24/279,998 | 1.26 (0.75, 2.12) | 0.387 | 24/281,312 | 1.22 (0.72, 2.04) | 0.460 |
| Unfavorable lifestyle | 27/188,757 | 2.30 (1.37, 3.86) | 0.002 | 31/187,851 | 2.32 (1.43, 3.77) | 0.001 | 32/188,765 | 2.28 (1.41, 3.69) | 0.001 |
| Intermediate genetic risk | |  |  |  |  |  |  |  |  |
| Favorable lifestyle | 150/1,620,096 | 1.60 (1.08, 2.35) | 0.018 | 162/1,613,772 | 1.53 (1.06, 2.20) | 0.023 | 165/1,620,117 | 1.52 (1.06, 2.17) | 0.024 |
| Intermediate lifestyle | 115/836,151 | 2.28 (1.53, 3.40) | <0.001 | 123/832,211 | 2.16 (1.48, 3.15) | <0.001 | 124/836,165 | 2.10 (1.45, 3.05) | <0.001 |
| Unfavorable lifestyle | 102/566,679 | 2.86 (1.91, 4.29) | <0.001 | 119/563,501 | 2.95 (2.01, 4.31) | <0.001 | 119/566,705 | 2.81 (1.93, 4.09) | <0.001 |
| High genetic risk | |  |  |  |  |  |  |  |  |
| Favorable lifestyle | 75/539,454 | 2.38 (1.57, 3.62) | <0.001 | 84/537,556 | 2.36 (1.59, 3.51) | <0.001 | 85/539,469 | 2.33 (1.58, 3.44) | <0.001 |
| Intermediate lifestyle | 57/278,409 | 3.39 (2.19, 5.25) | <0.001 | 60/276,886 | 3.17 (2.08, 4.81) | <0.001 | 61/278,415 | 3.10 (2.05, 4.69) | <0.001 |
| Unfavorable lifestyle | 54/188,287 | 4.55 (2.92, 7.10) | <0.001 | 61/187,406 | 4.54 (2.99, 6.90) | <0.001 | 61/188,296 | 4.33 (2.86, 6.56) | <0.001 |
| **Subgroup** | **Unweighted lifestyle categories (n=429,515)** | | | **Weighted lifestyle categories, multivariate imputed (n=429,515)** | | | **Participants with incomplete data of covariables excluded (n=425,523)** | | |
|  | **Events/Person-years** | **HR (95% CI)** | ***P* value** | **Events/Person-years** | **HR (95% CI)** | ***P* value** | **Events/Person-years** | **HR (95% CI)** | ***P* value** |
| Low genetic risk | |  |  |  |  |  |  |  |  |
| Favorable lifestyle | 36/537,238 | 1 (Ref) |  | 36/537,238 | 1 (Ref) |  | 36/533,017 | 1 (Ref) |  |
| Intermediate lifestyle | 24/281,312 | 1.22 (0.73, 2.05) | 0.450 | 24/281,312 | 1.22 (0.73, 2.05) | 0.450 | 24/278,378 | 1.22 (0.73, 2.05) | 0.449 |
| Unfavorable lifestyle | 32/188,765 | 2.32 (1.44, 3.74) | 0.001 | 32/188,765 | 2.32 (1.44, 3.74) | 0.001 | 32/186,460 | 2.33 (1.44, 3.75) | 0.001 |
| Intermediate genetic risk | |  |  |  |  |  |  |  |  |
| Favorable lifestyle | 165/1,620,117 | 1.51 (1.06, 2.17) | 0.024 | 165/1,620,117 | 1.51 (1.06, 2.17) | 0.024 | 163/1,606,544 | 1.50 (1.04, 2.15) | 0.029 |
| Intermediate lifestyle | 124/836,165 | 2.11 (1.46, 3.06) | <0.001 | 124/836,165 | 2.11 (1.46, 3.06) | <0.001 | 124/827,234 | 2.11 (1.46, 3.07) | <0.001 |
| Unfavorable lifestyle | 119/566,705 | 2.85 (1.96, 4.15) | <0.001 | 119/566,705 | 2.85 (1.96, 4.15) | <0.001 | 119/560,155 | 2.85 (1.96, 4.15) | <0.001 |
| High genetic risk | |  |  |  |  |  |  |  |  |
| Favorable lifestyle | 85/539,469 | 2.33 (1.58, 3.44) | <0.001 | 85/539,469 | 2.33 (1.58, 3.44) | <0.001 | 85/535,005 | 2.33 (1.58, 3.44) | <0.001 |
| Intermediate lifestyle | 61/278,415 | 3.12 (2.06, 4.71) | <0.001 | 61/278,415 | 3.12 (2.06, 4.71) | <0.001 | 60/275,735 | 3.06 (2.03, 4.64) | <0.001 |
| Unfavorable lifestyle | 61/188,296 | 4.40 (2.91, 6.66) | <0.001 | 61/188,296 | 4.40 (2.90, 6.66) | <0.001 | 59/186,279 | 4.25 (2.80, 6.46) | <0.001 |

CI indicates confidence interval; HR, hazard ratio.

Supplementary Table 10. Risk of Incident ulcerative colitis by genetic and lifestyle risk after excluding incident Crohn’s disease within 2 years after baseline, after excluding baseline colorectal cancer, additionally adjusted for depression, with unweighted lifestyle score, weighted lifestyle score in multivariate imputed data, and after excluding participants with incomplete data of covariables

| **Subgroup** | **Incident UC within 2 years after baseline excluded (n=430,247)** | | | **Prevalent colorectal cancer at baseline excluded (n=428,298)** | | | **Additionally adjusted for depression (n=430,384)** | | |
| --- | --- | --- | --- | --- | --- | --- | --- | --- | --- |
|  | **Events/Person-years** | **HR (95% CI)** | ***P* value** | **Events/Person-years** | **HR (95% CI)** | ***P* value** | **Events/Person-years** | **HR (95% CI)** | ***P* value** |
| Low genetic risk | |  |  |  |  |  |  |  |  |
| Favorable lifestyle | 78/538,400 | 1 (Ref) |  | 83/536,380 | 1 (Ref) |  | 84/538,409 | 1 (Ref) |  |
| Intermediate lifestyle | 79/332,764 | 1.53 (1.12, 2.09) | 0.008 | 82/331,266 | 1.49 (1.10, 2.02) | 0.010 | 83/332,770 | 1.49 (1.10, 2.02) | 0.010 |
| Unfavorable lifestyle | 38/138,298 | 1.61 (1.09, 2.38) | 0.016 | 44/137,500 | 1.77 (1.23, 2.54) | 0.002 | 45/138,308 | 1.78 (1.23, 2.56) | 0.002 |
| Intermediate genetic risk | |  |  |  |  |  |  |  |  |
| Favorable lifestyle | 324/1,617,444 | 1.38 (1.08, 1.77) | 0.010 | 352/1,611,648 | 1.39 (1.10, 1.77) | 0.006 | 352/1,617,488 | 1.39 (1.10, 1.77) | 0.006 |
| Intermediate lifestyle | 325/996,418 | 2.10 (1.64, 2.69) | <0.001 | 349/991,329 | 2.11 (1.66, 2.68) | <0.001 | 353/996,460 | 2.11 (1.66, 2.68) | <0.001 |
| Unfavorable lifestyle | 183/411,491 | 2.61 (2.00, 3.41) | <0.001 | 205/409,210 | 2.70 (2.09, 3.49) | <0.001 | 205/411,522 | 2.72 (2.10, 3.51) | <0.001 |
| High genetic risk | |  |  |  |  |  |  |  |  |
| Favorable lifestyle | 155/537,790 | 1.99 (1.51, 2.61) | <0.001 | 169/535,689 | 2.05 (1.58, 2.66) | <0.001 | 172/537,814 | 2.05 (1.58, 2.66) | <0.001 |
| Intermediate lifestyle | 152/330,975 | 2.96 (2.25, 3.89) | <0.001 | 168/329,118 | 3.06 (2.35, 3.97) | <0.001 | 169/331,001 | 3.05 (2.35, 3.97) | <0.001 |
| Unfavorable lifestyle | 105/138,553 | 4.46 (3.32, 6.00) | <0.001 | 113/137,854 | 4.43 (3.33, 5.89) | <0.001 | 113/138,566 | 4.46 (3.35, 5.93) | <0.001 |
| **Subgroup** | **Unweighted lifestyle categories (n=430,384)** | | | **Weighted lifestyle categories,** **multivariate imputed (n=430,384)** | | | **Participants with incomplete data of covariables excluded (n=426,377)** | | |
|  | **Events/Person-years** | **HR (95% CI)** | ***P* value** | **Events/Person-years** | **HR (95% CI)** | ***P* value** | **Events/Person-years** | **HR (95% CI)** | ***P* value** |
| Low genetic risk | |  |  |  |  |  |  |  |  |
| Favorable lifestyle | 90/538,340 | 1 (Ref) |  | 84/538,409 | 1 (Ref) |  | 81/533,207 | 1 (Ref) |  |
| Intermediate lifestyle | 61/280,619 | 1.20 (0.87, 1.66) | 0.272 | 83/332,770 | 1.49 (1.10, 2.02) | 0.010 | 83/329,756 | 1.54 (1.14, 2.10) | 0.006 |
| Unfavorable lifestyle | 61/190,527 | 1.64 (1.18, 2.28) | 0.003 | 45/138,308 | 1.77 (1.23, 2.55) | 0.002 | 45/136,364 | 1.85 (1.28, 2.66) | 0.001 |
| Intermediate genetic risk | |  |  |  |  |  |  |  |  |
| Favorable lifestyle | 372/1,622,072 | 1.37 (1.09, 1.72) | 0.007 | 352/1,617,488 | 1.39 (1.10, 1.77) | 0.006 | 347/1,603,140 | 1.42 (1.12, 1.81) | 0.004 |
| Intermediate lifestyle | 284/838,688 | 1.86 (1.47, 2.37) | <0.001 | 353/996,460 | 2.11 (1.66, 2.68) | <0.001 | 348/987,264 | 2.16 (1.69, 2.75) | <0.001 |
| Unfavorable lifestyle | 254/564,710 | 2.31 (1.81, 2.94) | <0.001 | 205/411,522 | 2.71 (2.10, 3.50) | <0.001 | 205/406,626 | 2.82 (2.17, 3.65) | <0.001 |
| High genetic risk | |  |  |  |  |  |  |  |  |
| Favorable lifestyle | 179/538,823 | 1.98 (1.54, 2.56) | <0.001 | 172/537,814 | 2.05 (1.58, 2.66) | <0.001 | 172/533,402 | 2.12 (1.63, 2.76) | <0.001 |
| Intermediate lifestyle | 137/278,418 | 2.72 (2.08, 3.55) | <0.001 | 169/331,001 | 3.06 (2.35, 3.97) | <0.001 | 165/327,558 | 3.09 (2.37, 4.04) | <0.001 |
| Unfavorable lifestyle | 138/190,140 | 3.73 (2.86, 4.88) | <0.001 | 113/138,566 | 4.45 (3.35, 5.91) | <0.001 | 110/137,246 | 4.49 (3.36, 5.99) | <0.001 |

CI indicates confidence interval; HR, hazard ratio.


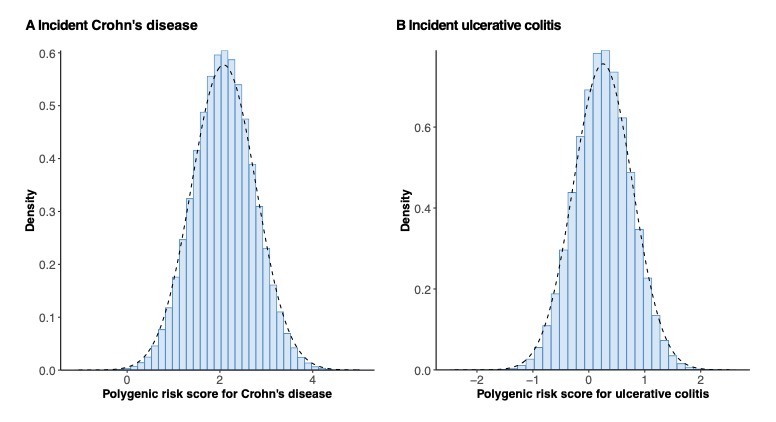


Supplementary Figure 1. Distribution of the polygenic risk score for Crohn’s disease (A) and ulcerative colitis (B).
